# Antibody binding of amyloid beta peptide epimers/isomers and ramifications for immunotherapies and drug development

**DOI:** 10.1038/s41598-023-38788-1

**Published:** 2023-07-31

**Authors:** Elizabeth R. Readel, Arzoo Patel, Joshua I. Putman, Siqi Du, Daniel W. Armstrong

**Affiliations:** Department of Chemistry and Biochemistry, The University of Texas at Arlington, Arlington, TX 76019 USA

**Keywords:** Proteins, Bioanalytical chemistry, Liquid chromatography, Protein purification

## Abstract

Extracellular deposition of amyloid beta (Aβ) peptide is a contributing factor of Alzheimer’s disease (AD). Considerable effort has been expended to create effective antibodies, or immunotherapies, targeting Aβ peptides. A few immunotherapies are thought to provide some benefit. It is possible that a contributing factor to the responses of such therapies may be the presence of modified, or aberrant, Aβ peptides found in AD patients. These aberrations include the isomerization and epimerization of L-Asp and L-Ser residues to form D-Asp, L/D-isoAsp, and D-Ser residues, respectively. An effective methodology is essential to isolate all Aβ peptides and then to quantify and locate the aberrant amino acids. Modifications to Aβ peptides may elevate the deposition of Aβ plaques and/or contribute to the neurodegeneration in AD patients, and may alter the binding affinity to antibodies. Herein, we used immunoprecipitation to examine the binding affinity of four antibodies against 18 epimeric and/or isomeric Aβ peptides compared to wild type (all L) Aβ peptide. Tandem mass spectrometry was used as a detection method, which also was found to produce highly variable results for epimeric and/or isomeric Aβ.

## Introduction

The misfolding and deposition of amyloid beta (Aβ) peptides into aggregates is associated with the progression and diagnosis of Alzheimer’s disease (AD)^1–3^. Aβ peptides commonly have 37–42 amino acids, with Aβ 1–42 as the predominant form in the brain^2^. However, aberrant types of Aβ peptides have been detected, which may include point mutations at various residues, in addition to racemization and/or isomerization of Asp and Ser residues^4^. It has been suggested that such variations contribute to and enhance neuronal toxicity as unique modifications change the spatial conformation and also hinder enzymatic degradation of Aβ^5–9^. Furthermore, Aβ peptides can form varying higher order structures such as insoluble extracellular plaques or soluble oligomeric species^10^. These higher order structures compromise targeting efforts, such as immunotherapies which rely on antibody binding efficacy to extract Aβ from AD brain^11^. Note that antibodies target peptide regions, known as epitopes, that are specific linear segments of amino acids. However, some antibodies specifically target spatial conformations, or higher order structures of a peptide. Rarely do antibodies have epitope regions that select for both a specific, linear segment and a higher-ordered spatial conformation^12^. Thus, the combination of aberrant Aβ species, and their subsequent unique solubilities and spatial conformations, add further challenges to developing effective antibodies targeting all Aβ peptide sequences.

Immunoprecipitation (IP) is typically used to purify and concentrate peptides from challenging matrices and can be used to evaluate the binding efficacy of an antibody to epimeric and/or isomeric proteins^13^. IP relies on the binding efficacy of an antibody-antigen to an agarose support. The elution product will consist only of the antigens that are selected for by the antibody^14^. Thus, the epimers and/or isomers can be compared to their natural all L-antipode.

Interestingly, commercial antibodies targeting Aβ, and thus immunotherapies, exclusively target unmodified Aβ peptides (i.e. the wild-type (WT) all L-Aβ peptide)^11,15^. It is assumed that WT (all L) Aβ contains no isomeric and/or epimeric centers. No published work has considered whether these antibodies also target epimeric and/or isomeric Aβ peptides. Interestingly, there are a few studies that have shown a negative effect on antigen binding when an iso-Asp residue is located in, or near, the binding region of certain antibodies^16–18^. Thus, one should consider altered antibody binding affinities to antigens or peptides that have isomerized and/or racemized amino acids. Further, these aberrations may have consequences when developing immunotherapies^19^. Evidence shows that Aβ peptide can easily isomerize and/or epimerize in at least five locations including Asp1, Asp7, Asp23, Ser8 and Ser26 in AD brain. It has been reported that at least 20% of Asp1 and 75% of Asp7 of Aβ are L/D-iso-Asp in AD brain samples, compared to 6% of Asp1 in non-AD Aβ brain samples^7^. Additionally, 4–9% of Ser residues in AD brain are D-Ser^9^. Research has shown elevated levels of L-iso-Asp, D-iso-Asp and D-Asp in older subjects as compared to younger subjects, suggesting that the conversion of L-Asp to its succinimide antipodes is a time-dependent hallmark of aging^7^. Therefore, to better target the diverse catalogue of possible epimeric and/or isomeric Aβ peptides as an analytical methodology and possibly as an immunotherapy, it is critical to understand how antibody binding efficacies may be disrupted, or possibly enhanced, for modified/aberrant peptides.

**Figure 1:**
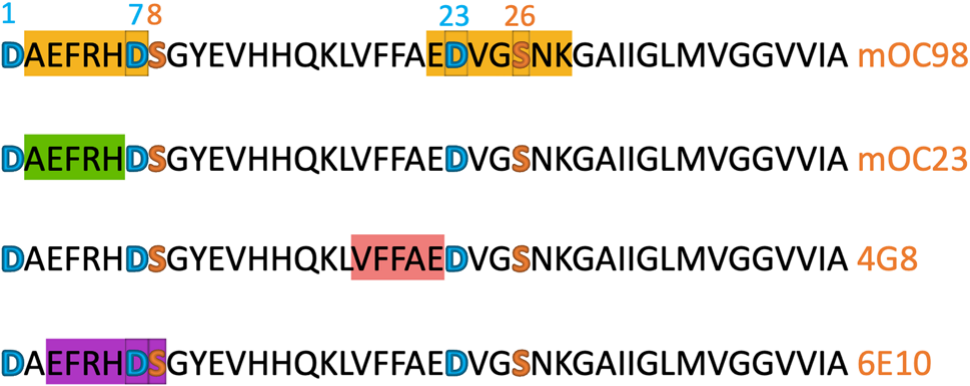
Epitope binding region of the four antibodies used in this study, with names indicated on the right in orange. The epitope binding region is highlighted on the Aβ 1–42 sequence for each antibody. The aberrant aspartic acid residues are in blue while the aberrant serine residues are in orange.

Antibodies 6E10 and 4G8 are among the first monoclonal antibodies commercially available that target Aβ 1–42^20,21^. They are commonly used in immunoprecipitation, immunohistochemistry, and Western blotting to measure or identify Aβ from biological matrices^22–24^. A high-resolution mapping technique identified the epitope region for 6E10 to Aβ residues 4–10 and for 4G8 to Aβ residues 18–23 (Fig. 1)^25^. Unlike antibodies 4G8 and 6E10, which recognize linear segments of Aβ 1–42, OC-type monoclonal antibodies recognize conformational epitopes and prefer amyloid aggregates over monomers^12^. Antibodies mOC98 and mOC23 have N-terminal epitope binding regions (Fig. 1). While they are classified as having conformational epitopes, they also are known to be reactive to monomeric Aβ 1–42^26^. It should be noted that the aforementioned antibodies were developed using the WT (all L) Aβ peptide as it was assumed to be the only naturally occurring form. To obtain analytically and/or biologically relevant results, it is crucial to understand antibody affinity to WT (all L) versus aberrant Aβ peptides.

An additional consideration for the analysis of epimeric and/or isomeric peptides are their detection by MS/MS. Epimeric and/or isomeric peptides can have unique solubilities and spatial conformations in solution which may affect the instrument response when analyzed by MS/MS^27^. In addition, epimers and/or isomers may have preferred ionization charge states and fragmentation pathways, thereby affecting their MS/MS intensities^28–30^. Effectively, this may affect the quantitative analysis of epimeric and isomeric peptides when using the same MS/MS conditions.

The goal of this study was to investigate antibody binding of WT (all L) Aβ 1–42 versus its epimers and/or isomers and to determine the effect, if any, of such stereochemical aberrations. To accomplish this, 18 epimers and/or isomers of Aβ with single- and double-point mutations were screened against four antibodies. These 18 epimers/isomers had aberrations including L-iso-Asp, D-iso-Asp, D-Asp, and D-Ser modifications at positions Asp1, Asp7, Asp23, Ser8 and Ser26, respectively. The four screened antibodies include 4G8, 6E10, mOC98, and mOC23.

## Results

### Antibody and aberrant Aβ selection

**Figure 2:**
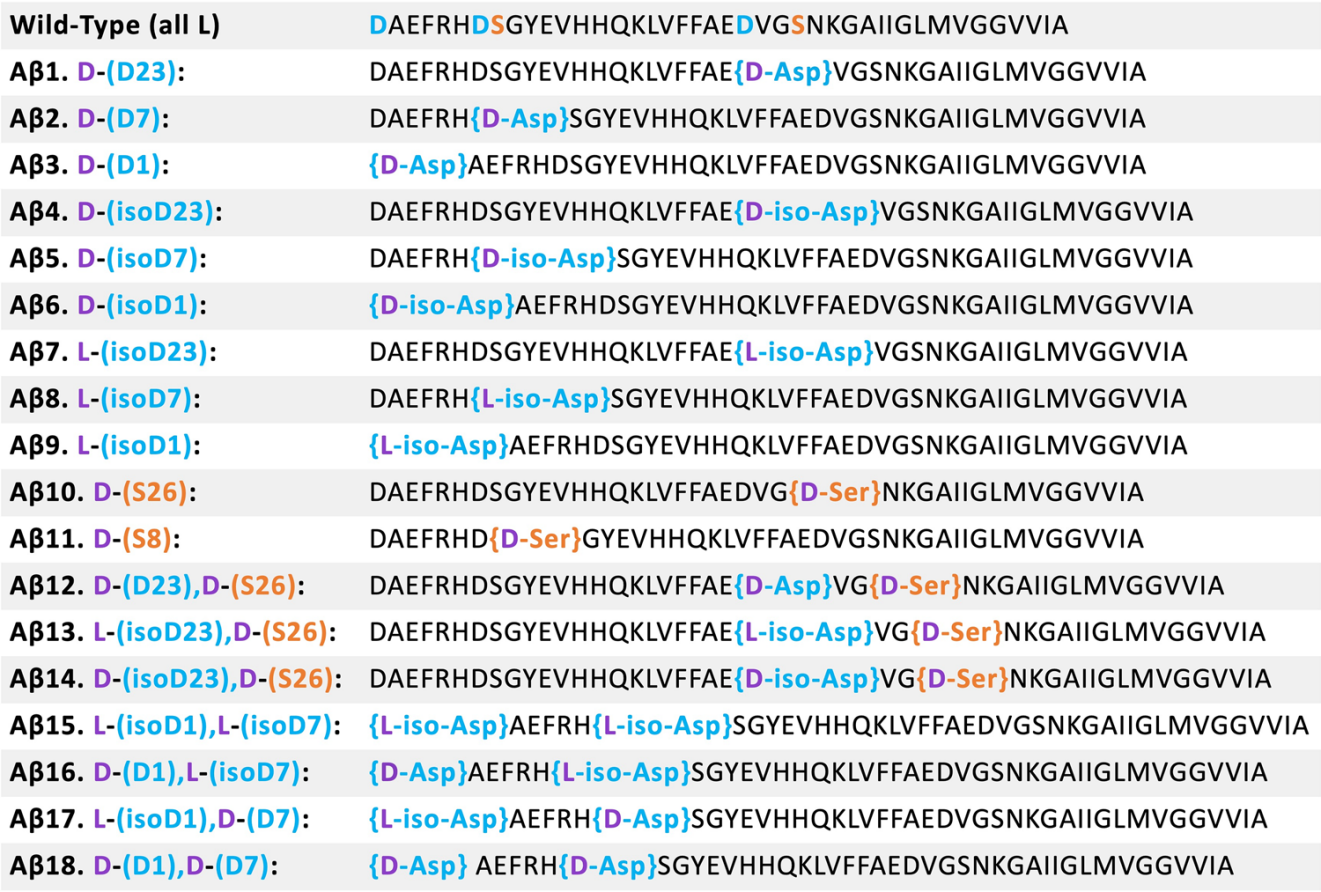
List of Aβ peptide isomers and/or epimers assessed in this study, with the aberrations indicated in brackets. The peptides are labeled with the first letter indicating the stereochemical configuration (i.e., L,D) (in purple) and the amino acid residue and position in parenthesis. The aberrant aspartic acid residues are in blue while the aberrant serine residues are in orange.

A selection of antibodies was chosen to examine the effect of antigen binding across several epitope regions of the WT (all L) and aberrant Aβ 1–42. As can be seen, the antibody epitopes specifically targets Asp and Ser positions, and/or directly adjacent regions (Fig. 1). These antibodies include 4G8, 6E10, mOC98, and mOC23. Note that antibody mOC98 has two epitope binding regions which accommodate amino acids Asp7, Asp23, and Ser26. Antibody mOC23 does not overlap with any Asp or Ser residues, but is encompassed by Asp1, Asp7 and Ser8. The four antibodies were screened against all Aβ peptides listed in Fig. 2. The aberrant Aβ 1–42 peptides are listed below the WT (all L) peptide. To address whether the antibody epitope region was specific to the configuration of Asp or Ser residues, peptides with single mutations for all possible Asp modifications and Ser were screened. These peptides correspond to Aβ1–Aβ11 (see Fig. 2). It is likely that more than one modification will be present on Aβ 1–42 extracted from AD brain. To address this possibility, a selection of doubly modified peptides was analyzed. Note that the double modifications are at positions Asp23 and Ser26 or Asp1 and Asp7. Double modifications at positions Asp23 and Ser26 were chosen as these amino acids are in the core of Aβ 1–42. The amino acids in the middle of Aβ 1–42 contribute to higher order structures^10^. In contrast, double modifications at positions Asp1 and Asp7 were selected as these amino acids are in the region that binds many commercial antibodies. In addition, Aβ 1–42 extracted from AD brain was found to have significant modifications at positions Asp1 and Asp7. These “aberrant” peptides correspond to Aβ12–Aβ18 (Fig. 2).

### Antibody-antigen binding affinities by immunoprecipitation

**Figure 3:**
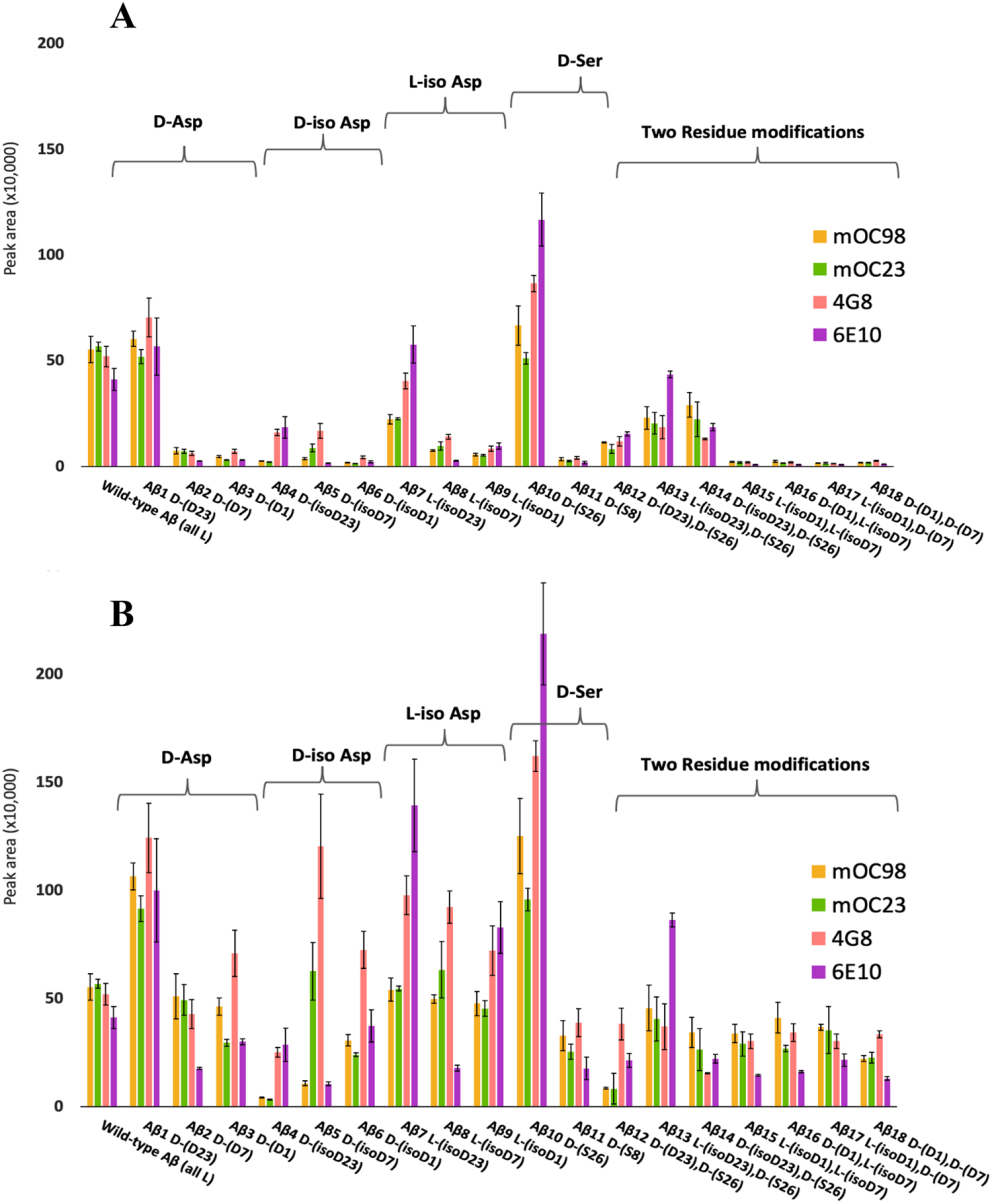
(**A**) shows resulting peak areas of immunoprecipitation extraction of 18 peptides with four antibodies (see above figures for details). (**B**) shows the data from (**A**) after corrections for sensitivity of MS/MS detection for each peptide. The peptides are labeled according to Fig. 2.

Antibody binding efficacy was assessed through an immunoprecipitation procedure, followed by reconstitution in borate buffer and analysis via liquid chromatography tandem mass spectrometry (see Methods). The subsequent MS/MS peak areas were integrated, and their intensity areas were plotted as seen in Fig. 3A. Note that these antibodies were developed using the WT (all L) Aβ peptide. Further, the MS/MS detection also was optimized using the WT (all L) Aβ peptide. The antigen binding for the WT (all L) Aβ was similar for all antibodies (4G8, 6E10, mOC98 and mOC23) which was not unexpected. However, it also is apparent (Fig. 3A) that the signal intensities for all but two of the “aberrant” peptides (i.e. Aβ1 D-(D23) and Aβ10 D-(S26)) are far lower than those of the WT (all L) Aβ peptide., the peptides with epimerizations or isomerization near the N-terminus of the Aβ usually exhibited signal intensities approximately 10–50 times lower than the WT (all L) Aβ peptide. The reduced signal intentensities could be attributed to the altered binding between the antibody and the “aberrant” forms of Aβ, differences in the MS/MS of the “aberrant” Aβ or combinations of these two factors.

In order to ascertain whether or not the presence of aberrant amino acids affected the selectivity of mass spectrometry, calibration curves for each aberrant peptide were created using the MS/MS conditions optimized for the WT (all L) Aβ (see Supplementary Table S1). The slopes for each aberrant peptide varied from the slope of the WT (all L) Aβ when using the same instrument conditions, and in some cases, quite significantly. Therefore, the data of Fig. 3A was corrected with the individual calibration curves to reveal the correct binding affinities, as shown in Fig. 3B. Among the peptides with single modifications, a few showed binding affinities that are higher than those of the WT Aβ 1–42. For Aβ1 D-(D23) and Aβ10 D-(S26), every respective antibody extraction is higher than the WT (all L) Aβ antibodies. In contrast, Aβ2 D-(D7), Aβ4 D-(isoD23) and Aβ11 D-(S8) have lower binding affinities for all screened antibodies. In most cases, two or three of the four screened antibodies extracted single aberration peptides comparably to the WT (all L) Aβ. Additionally, in all but three cases (i.e. Aβ1 D-(D23), Aβ10 D-(S26) and Aβ7 L-(isoD23)) between one to four antibodies extracted significantly less of the aberrant Aβ peptides.

There was no discernible pattern indicating the success of antibody binding based off the type of modification or the modification location, either within or near an epitope region. In general, antibody 4G8 consistently appeared to provide the highest, or second highest, binding efficacy, except for Aβ2 D-(D7). All peptides with double modifications had binding affinities that were lower than the WT (all L) Aβ, except for Aβ13 L-(isoD23), D-(S26), in which only antibody 6E10 comparably extracted this epimer. For the peptides with double modifications at the N-terminus, peptides Aβ15–Aβ18, antibody 6E10 consistently had the lowest binding affinity. In contrast, peptides with double modifications nearer the middle of Aβ, at Asp23 and Ser26, did not show this trend. Overall, peptides with single modifications tended to have higher binding affinities than peptides with double modifications.

### Variability in MS/MS for epimers/isomers

**Figure 4:**
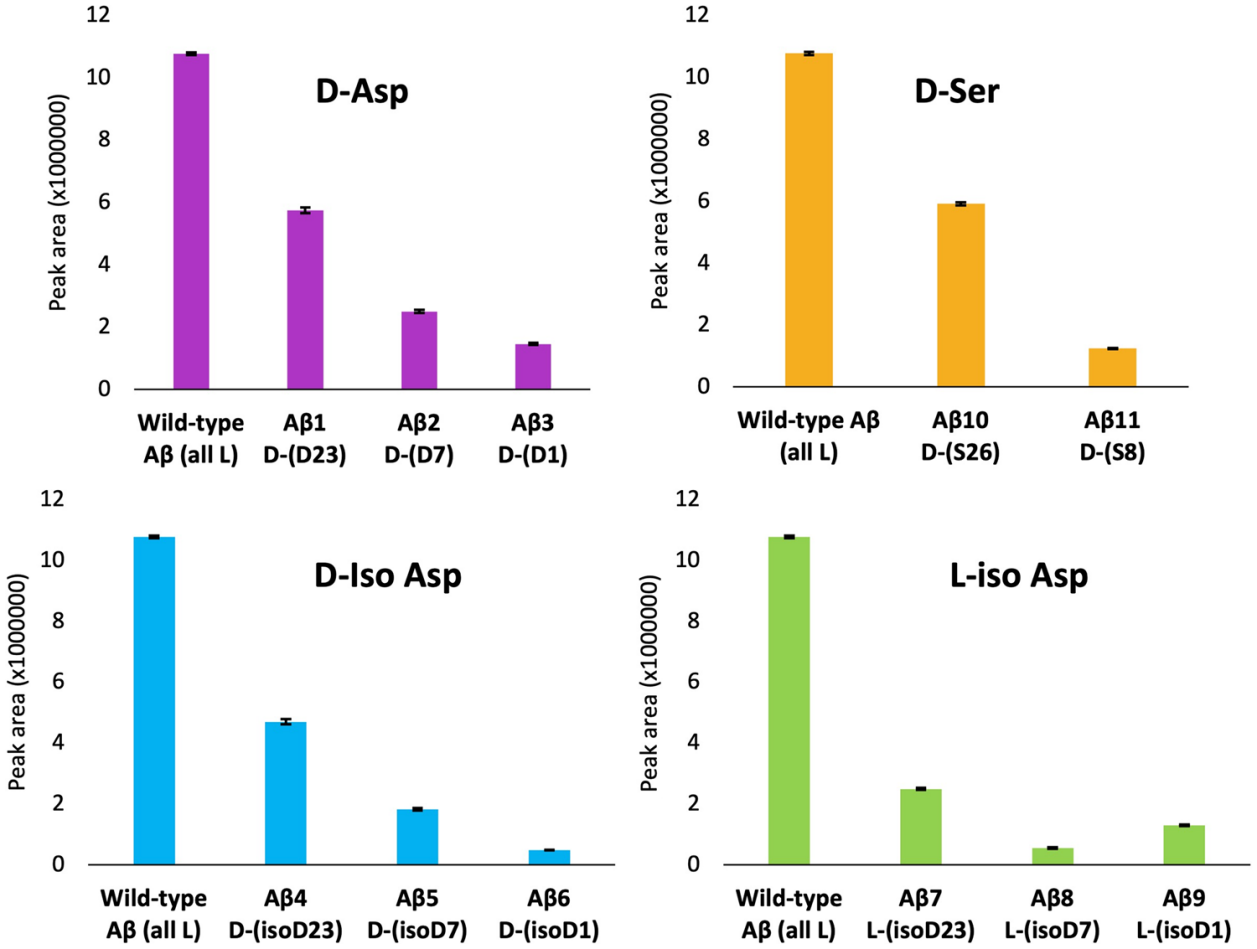
Peak areas of various peptides at 20 µg/mL concentration under the same MS/MS conditions (see Method). The WT (all L) peptide is plotted along with group of peptides with specified aberrations in each category (D-Asp, D-ser, D-iso Asp, L-iso Asp). Note, any epimeric or isomeric peptide is less sensitive than the WT (all L) peptide under the optimized conditions for the WT (all L) peptide.

Data trends in Fig. 3A, B contrast because Fig. 3A data has not been corrected to consider how MS/MS specificity may be affected by aberrations in peptides. To further highlight the differences in MS/MS specificity, the integrated peak area for the 20 µg/mL standard of each peptide is plotted in Fig. 4. Each type of Asp and Ser modifications are individually plotted against their positions in the Aβ peptide. The peak area, or sensitivity, decreases as the modification proceeds from the core region of Aβ to the N-terminus of Aβ. The sensitivity does not appear to depend on the type of modification. This includes the peptides with double modifications. Peptides with double modifications in the core region of Aβ exhibit higher MS/MS sensitivity than peptides with double modifications at the N-terminus under the same instrument conditions.

## Discussion

We have shown that isomeric and/or epimeric peptides of Aβ 1–42 have different binding affinities for antibodies 4G8, 6E10, mOC98, and mOC23 by immunoprecipitation. This selection of antibodies has diverse epitope regions which allows a better understanding of antibody–Aβ binding affinities. As expected, all four antibodies associated with the unmodified WT (all L) Aβ with the same efficacy as each antibody was manufactured using an unmodified, WT (all L) Aβ peptide. For this reason, WT (all L) Aβ served as a standard reference for the epimers and/or isomers.

Figure 3A versus B highlights the discrepancies that can occur with improper mass spectrometry data collection. Both figures depict the same dataset, but Fig. 3B corrects for the effect of MS/MS variability. Our studies reveal that epimers and/or isomers can have different sensitivities when using the same MS/MS conditions and that many epimeric and isomeric peptides are less sensitive. The secondary structure of Aβ monomers may be responsible for the reduced signal for epimeric and/or isomeric peptides. The secondary structures of Aβ monomers have alpha helical structures located near the core of the peptide, encompassing amino acids 8–25 to 28–38^10^ (which may also inhibit or enhance antibody binding capabilities). Our group has previously shown that aberrant Aβ peptides have different retention times when chromatographically separated using reversed-phase liquid chromatography^27^. This effect results from unique on-column interactions due to structural differences induced by peptide modifications between epimers and/or isomers. The unique secondary structures could lead to fragmentation differences during MS/MS analysis, and thereby affect the sensitivity of the peptide epimers and/or isomers when using the same MS/MS conditions. Interestingly, as seen in Fig. 4, the locations of the Aβ modification seems to affect the sensitivity more than the nature of the modification. Specifically, epimeric or isomeric centers near the N-terminus of Aβ had the biggest effects. This is the first investigation of peptide epimers and/or isomers that highlights the changes in MS/MS specificity based on the aberration location within a peptide. Nevertheless, we can easily correct for MS/MS specificity caused by peptide aberrations by creating calibration curves for each aberrant peptide at the same MS/MS conditions. Hence, the dataset in Fig. 3B best reflects the binding affinities of the antibodies to the aberrant Aβ peptides, and all further discussion of the results focuses on the Fig. 3B data.

Antibodies 4G8 and 6E10 were typically the most effective antibodies at extracting aberrant Aβ peptides, especially with epimers and/or isomers with core-amino acid modifications. These antibodies have especially high binding affinities for Aβ7 L-(isoD23), Aβ1 D-(D23) and Aβ 10 D-(S26). For all antibodies tested, Aβ1 D-(D23) and Aβ10 D-(S26) had higher antibody binding affinities than the WT (all L) Aβ. Note that Aβ4 D-(isoD23) had poor binding for all screened antibodies. Antibodies 4G8 and 6E10 are epitope sequence dependent while antibodies mOC98 and mOC23 are conformation dependent. It is evident that the epimers and/or isomers must have different conformations as their binding affinities for mOC98 and mOC23 vary dramatically, and rarely did an mOC-based antibody bind preferentially in comparison to 4G8 and/or 6E10. Also, there was no specific epitope that was predictive as to the success of an antibody-antigen pairing. Rather, Aβ peptides with modifications located in the middle of the peptide generally had a higher binding efficacy, independent of the antibody epitope region.

Single amino acid modifications were individually assessed for all possible forms of Asp and Ser, at all locations. There were no observable patterns regarding which antibody outperformed the others for Aβ peptides with single amino acid modifications (aberrations). However, the relative selectivity of the four tested antibodies for any Aβ with a single amino acid aberration could be 2–10 times different in most cases. Furthermore, the location of the amino acid aberration within Aβ had a significant effect on binding effectiveness. Specifically, antibody binding affinities decreased when the epimer and/or isomeric amino acid was near the N-terminus of Aβ. The most dramatic example of this effect is for Aβ9 D-(S8) versus Aβ10 D-(S26). Collectively, Aβ10 D-(S26) had the highest combined selectivity for the antibodies while Aβ11 D-(S8) had a very low recovery, as observed for Aβ with double modifications. Peptides with D-Asp and L-iso-Asp also share this effect: the same modification at the core of Aβ has generally higher antibody binding than modifications located near the N-terminus. Aβ peptides with a D-iso-Asp modification are the exception to this trend. The differences in binding are attributed to structural changes induced by the modification. This could explain why several of the aberrant peptides have higher binding than the unmodified WT (all L) Aβ 1–42, while many others had significantly lower binding. Two-residue modifications most consistently had the lowest binding affinity of all the peptides screened. In contrast to the singly modified peptides, the double modifications located at the core of Aβ do not necessarily have higher binding than modifications at the N-terminus. It appears that having multiple modifications increases the possibility for unique peptide spatial conformations which, in turn, inhibits antibody binding. Thus, it was generally observed that Aβ with two amino acid modifications did not bind as effectively as the WT (all L) Aβ.

In a few cases, aberrant Aβ peptides may induce more favorable antibody-antigen interactions, or rather, stabilize the most complimentary spatial arrangement of an antigen. Isomeric and/or epimeric Aβ monomers can form aggregates adding to their unique spatial conformations. A combination of complimentary factors may induce antibody-antigen binding, such as unique monomer secondary structure, or formation of small oligomers. Note that the WT (all L) Aβ peptide gradually forms oligomers, then protofibrils, and eventually fibrils. This rate dependent formation should be unique for the aberrant species, leading to unique binding capabilities of higher order structures. Our results suggest that utilizing a cocktail of antibodies that include linear and conformation specific epitopes would ensure a more comprehensive extraction of all Aβ peptides. However, this may not be a plausible solution for immunotherapies targeting Aβ peptides in Alzheimer’s Disease patients.

Antibodies are being tested and used as AD drugs and a significant factor contributing to their efficacy is directly related to their binding affinities to the Aβ peptide. These immunotherapies have been in development for over a decade, and several have completed clinical trials and proceeded to marketing^31,32^. However, these immunotherapies are not a cure-all for Alzheimer’s disease. In fact, they are targeted to slow the progression of AD in patients. A myriad of reasons could explain their lack of success including some failures in clinical trials occurring after AD has progressed too much. However, current immunotherapies are assumed to use antibodies that targe the WT (all L) Aβ peptide. While, a significant amount of Aβ extracted from AD brain has aberrations located at the N-terminus–which, as shown, can be a challenging epitope location for antibody interactions. Although, we were able to achieve some binding affinities for all aberrant Aβ peptides screened, it is pertinent to note that most immunotherapies targeting Aβ peptide presumably have epitope regions that are highly selective to WT (all L) Aβ peptide and could be less likely to bind peptides with aberrations. Hence, it should be considered that such therapies may be less effective in vivo given the aberrations in Aβ peptides that have been reported in previous studies.

## Conclusions

Studies targeting aberrant Aβ peptides need to evaluate epimers and/or isomers as discrete peptides from their WT all L-antipodes. Indeed, epimers and/or isomers can have unique spatial conformations and/or aggregational behaviors which can affect antibody binding and change MS/MS ionization and/or fragmentation. For these reasons, epimers and/or isomers could impose considerable challenges when developing immunotherapies. Further investigations testing binding affinities of antibodies for marketed immunotherapies and those in various stages of clinical trials may clarify the actions and/or shortcomings of these therapies. It is also plausible that previous analyses that used antibodies to identify aberrant peptides in AD brain may have incorrectly reported the population of aberrant Aβ. Clearly, additional in-depth studies on therapeutic antibodies binding to aberrant Aβ and their MS/MS behaviors are warranted. Investigations of known immunotherapy antibodies and various forms of Aβ 1–42, in addition to tryptic digest fragments, are currently underway.

## Methods

### Antibody/antigen purification and extraction with Immunoprecipitation (IP)

WT (all L) Aβ 1–42 peptide was purchased from Genscript (Piscataway, NJ, USA) and the aberrant Aβ 1–42 synthetic peptides (> 95% purity) were purchased from Peptide 2.0 (Chantilly, VA, USA). Stock solutions of each Aβ standard were prepared in 10 mM borate buffered to pH 9.1 with sodium hydroxide. Boric acid, sodium hydroxide, formic acid and trifluoroacetic acid were obtained from Sigma Aldrich (St. Louis, MO, USA). Peptide concentrations were standardized using a Pierce™ BCA Protein Assay kit from Thermo Fisher Scientific (Waltham, MA, USA).

Purified anti-β-Amyloids 17–24 Antibody (4G8) and 1–16 Antibody (6E10) were purchased from BioLegend (San Diego, CA, USA) while mOC-type antibodies recombinant anti-beta amyloid 1–42 mOC98 and mOC23 were purchased from Abcam (Cambridge, UK). Antibody-antigen binding was measured using Pierce Classic IP Kit by Thermo Fisher Scientific (Waltham, MA, USA). Per IP experiment, 11 pmol Aβ peptide (2 µL of 25 ug/mL stock solution solution) and 13.2 pmol of antibody were added to 300 µL IP lysis/wash buffer and allowed to bind to form antigen–antibody complexes. Protein A/G plus agarose resin was added to couple with antibody-antigen complexes. Complexes were washed in IP lysis/wash buffer followed by conditioning buffer. Aβ peptides were immunoprecipitated using elution buffer. Elution buffer was evaporated and samples were reconstituted in 20 µL of 0.1 M borate buffered to pH 9.1 and analyzed by LC–MS/MS. All samples were prepared in triplicate.

### Data collection by liquid chromatography tandem mass spectrometry (LC–MS/MS)

HPLC–MS grade acetonitrile was purchased from Sigma-Aldrich and ultrapure water was obtained from a Milli-Q-water system (Millipore, Bedford, MA, USA). Samples were analyzed using an LCMS/MS-8050 (Shimadzu Scientific Instruments, Columbia, MD, USA), triple quadrupole spectrometer equipped with a positive ESI source and instrument conditions: drying gas and nebulizing gas flow rate of 15 and 2 L/min, respectively; desolvation line temperature and heat block temperature of 275 and 400 °C, respectively. WT (all L) Aβ 1–42 and aberrant Aβ 1–42 immunoprecipitated standards were quantitated using the MS/MS transition 903–886 m/z. The peptides were eluted using BIOshell™ IgG 1000 Å C4 column (4.6 mm i.d × 10 cm length, pore size 1000 Å, particle size 2.7 um; Supelco, Bellefonte, PA, USA). At a flow rate of 0.4mL/min, the elution conditions include a ramp from 5% mobile phase B (99.9% acetonitrile, 0.1% formic acid) and 95% mobile phase A (99.9% water, 0.1% formic acid) to 50% mobile phase B and 50% mobile phase A from 0 to 5 min, followed by an isocratic hold at 50% mobile phase B and 50% mobile phase A from 6 to 8 min, then a wash in 100% mobile phase C (50.0% water, 49.9% acetonitrile, 0.1% trifluoracetic acid) for 10 min, followed by a column reconditioning at 5% mobile phase B and 95% mobile phase A for 10 min. Shimadzu LabSolutions software was used to integrate the peak areas.

## Supplementary Information

Supplementary Information.

## Data Availability

The datasets generated during and/or analyzed during the current study are available from the corresponding author on reasonable request.
