# Supplementary material for: Antibody binding of amyloid beta peptide epimers/isomers and ramifications for immunotherapies and drug development
Source: Sci Rep. 2023 Jul 31;13:12387. doi: 10.1038/s41598-023-38788-1 (PMC10390520; doi:10.1038/s41598-023-38788-1)
Supplement: Supplementary file 1 — Supplementary Information. [file 41598_2023_38788_MOESM1_ESM.pdf]

Supplementary Information:

Antibody Binding of Amyloid Beta Peptide Epimers/Isomers: Ramifications for Immunotherapies and Drug Development

Elizabeth R. Readel, Arzoo Patel, Joshua I. Putman, Siqi Du and Daniel W. Armstrong*

Department of Chemistry and Biochemistry, The University of Texas at Arlington, Arlington, TX 76019, USA

Email: sec4dwa@uta.edu

1.1 Calibration curves for aberrant Aβ peptides

Calibration curves for each aberrant Aβ peptide was created with a range of 5 ug/mL to 100 ug/mL standards. Each standard was injected in triplicate (relative standard deviation < 10% for each sample) using the same MS/MS instrument conditions outlined in the Methods section. The conditions were optimized for the all-L Aβ peptide. The slopes for each peptide can be found in Table S1. The linearity for each curve had an R^2 of minimum 0.990. All aberrant Aβ peptides had smaller calibration curve slopes than the all L-Aβ peptide, indicating that they are less sensitive than the all L-Aβ while using the same instrument conditions. Additionally, the slopes for each aberrant peptide trended to decrease as the modification moved from the core of the peptide to the N-terminus. Further, double modified Aβ peptides typically had the least sensitivity.

Table S1. Slope and linearity for each aberrant peptide using the same MS/MS conditions. MS/MS conditions were optimized for the all L- Aβ peptide.

Peptide	Modification	Slope
All L-Aβ	-	454182
Aβ 1	D-(D23)	257686
Aβ 2	D-(D7)	65870
Aβ 3	D-(D1)	46024
Aβ 4	D-(isoD23)	293973
Aβ 5	D-(isoD7)	63435
Aβ 6	D-(isoD1)	26864
Aβ 7	L-(isoD23)	187802
Aβ 8	L-(isoD7)	68877
Aβ 9	L-(isoD1)	65870
Aβ 10	D-(S26)	242380
Aβ 11	D-(S8)	47055
Aβ 12	D-(D23), D-(S26)	140150
Aβ 13	L-(isoD23), D-(S26)	228670
Aβ 14	D-(isoD23), D-(S26)	384770
Aβ 15	L-(isoD1), L-(isoD7)	29485
Aβ 16	D-(D1), D-(isoD7)	27265
Aβ 17	L-(isoD1), D-(D7)	25600

Aβ 18	D-(D1), D-(D7)	36470
